# Comparative study of *Eimeria tenella* development in different cell culture systems

**DOI:** 10.1371/journal.pone.0307291

**Published:** 2024-07-18

**Authors:** Kelsilandia Aguiar-Martins, Fiona M. Tomley, Damer P. Blake, Virginia Marugan-Hernandez

**Affiliations:** The Royal Veterinary College, Department of Pathobiology and Population Sciences, Hawkshead Lane, University of London, London, United Kingdom; Penn State Health Milton S Hershey Medical Center, UNITED STATES OF AMERICA

## Abstract

Cell culture systems have long been recognised as great resources to mitigate the use of animals in research, offering effective solutions for replacement or reduction with benefits commonly including lower costs, shorter duration and improved reproducibility. The use of *in vitro* culture methods has been extensively explored for many apicomplexan parasites, supporting significant research advances, but studies with *Eimeria* are often limited since they still depend on the animal host. In this study we have used 2.5D and 3D culture systems for the first time to evaluate the growth of *Eimeria tenella* parasites using a panel of cell lines (MDBK, HD11, COLO-680N and HCC4006). Results were compared to growth in 2D monolayers following established protocols. Observations using the fluorescent transgenic strain Et-dYFP showed invasion and development of parasites inside cells suspended in a collagen matrix (2.5D or 3D), supporting the development of asexual stages with the release of first-generation merozoites. Similar findings were observed when Scaffold-free 3D cell spheroids of HD11 cells were infected with sporozoites. No subsequent developmental stages were identified while evaluating these cell lines and further work will be required to improve *in vitro* culture systems to a point where reduction and replacement of animal use becomes routine.

## 1. Introduction

*Eimeria tenella* is a common and pathogenic cause of caecal coccidiosis in domestic chickens. The parasite has a monoxenous life cycle that starts when the chicken ingests sporulated oocysts from the environment [1] The tough oocyst walls are broken within the crop and gizzard, releasing four sporocysts which each contain two haploid sporozoites. In the intestinal lumen sporozoites are stimulated to excyst from sporocysts and invade enterocytes of the caeca [2] within which they replicate asexually (schizogony) to produce daughter merozoites which are released to initiate the next round of infection. After usually three morphologically distinct generations of schizogony, invasion of the final merozoites results in the development and maturation of dimorphic male and female gametocytes [3]. The endogenous part of the life cycle is completed when the macrogametes are fertilised by motile microgametes and develop into diploid unsporulated oocysts that are released in faeces to sporulate in the environment [4].

Laboratory protocols for harvesting unsporulated oocysts and for controlled oocyst sporulation, breakage, excystation and sporozoite purification are well established for *E*. *Tenella* [5]. Purified sporozoites can invade at high efficiency and go through a single round of schizogony within immortalised epithelial cell lines, particularly Madin-Darby Bovine Kidney (MDBK) cells [6–9], but production of further asexual and sexual stages has not been achieved in these immortalised systems. The propagation of the entire *E*. *tenella* life cycle *in vitro* was described more than 50 years ago in primary chick kidney cells [10], but productivity beyond the first round of schizogony was extremely limiting and regular use of the model proved unsustainable. More recently a chicken cell line, CLEC-213, which has an epithelial cell phenotype, was shown capable of supporting development of gametocytes and oocysts following infection with second-generation merozoites of the Wisconsin strain of *E*. *Tenella* [11], but not with sporozoites of the same strain. Although this model has drawbacks, including the need to isolate infectious merozoites from chickens to initiate the *in vitro* infection, it could be a useful research tool for examining the detailed biology of the sexual cycle. However, the inability to propagate the *Eimeria* life cycle efficiently *in vitro* from sporozoites through to unsporulated oocysts has curtailed advances in Eimeria studies and resulted in the ongoing need for propagation of parasites in chickens [12].

Current *in vitro* systems for *Eimeria* cultivation are commonly based on two-dimensional (2D) cultures where cells form a monolayer adhered to a flat polystyrene surface modified to favour adhesion, employing a substrate not found in live organisms and that induces an artificial polarisation of the cells [13]. In 2D culture many essential cellular functions found *in vivo* are absent, including formation of the extracellular matrix, cell-to-cell and cell-to-matrix interactions [14]. Therefore, it is unsurprising that extrapolation of results from 2D culture systems to *in vivo* behaviours can be misleading, inaccurate or not possible [15, 16].

Three-dimensional (3D) cell culture is a way to potentially reduce the gap between 2D cultures and the cellular physiology of animals as these can more accurately represent the microenvironment where cells reside in tissues [17]. Several new methods are currently being explored to support 3D cultures, influenced by the complexity required to reproduce biological systems and the time and costs required for *in vivo* work. In collagen-based extracellular-like matrices (CBEM) cells are suspended in collagen gels, fibrin gels or a cell-derived matrix composed of proteins, that aims to reconstitute the physical and biological properties of the extracellular matrix [18]. These models favour the proliferation and differentiation of cells, allowing them to associate in a more physiologically relevant manner. In some cases, simplified methods using monolayers on top of collagen-based matrices (2.5D culture) have been explored to mitigate limitations associated with 3D cultures, such as cost and time required for the technique [19]. Spheroids are another 3D system which benefits from being simple to manipulate; they are non-adherent cell aggregates that allow cell-to-cell and cell-to-matrix interactions to occur, more accurately resembling the target microenvironment structure [20]. 3D cell culture systems have been used successfully to study apicomplexan parasites such as *Cryptosporidium* and *Toxoplasma gondii* [21, 22], but remarkably little progress has been reported for *Eimeria* [23]. Preliminary studies using chicken enteroids have suggested that *E*. *tenella* might be able to reach the macrogametocyte lifecycle stage in chicken caecal enteroids based upon detection of stage-specific gene transcription, although further development and validation is required [24].

In this study, we have evaluated infection and development of *E*. *tenella* in 2.5D and 3D cell cultures systems (collagen-based matrix and spheroids) using different cell lines, and compared them to the classical 2D system, aiming to achieve parasite development beyond first-generation merozoites in a readily accessible and reproducible culture system.

## 2. Materials and methods

### 2.1. Parasites

Infections were performed using a stable transgenic *E*. *tenella* Wisconsin population expressing a double cassette of the yellow fluorescent protein (Et-dYFP) [25]. These transgenic parasites were propagated in specific pathogen-free four-week-old Lohmann Valo chickens purchased from the Animal & Plant Health Agency, Weybridge, UK. Freshly-purified sporozoites used for cell culture infections were obtained following protocols detailed by [5].

### 2.2. Cells

We used four immortalised cell lines; two previously used to propagate *E*. *tenella* and two for which no prior data were available (Table 1). Each cell line was maintained in 75 cm^2^ treated culture flasks (Falcon) under specific conditions of incubation and media (Table 1) using either Advanced Dulbecco’s Modified Eagle’s Medium (DMEM; Gibco) or Roswell Park Memorial Institute (RPMI) 1640 Medium (Gibco) supplemented with 2% or 10% foetal bovine serum (FBS; Sigma), respectively, and 100 U penicillin/streptomycin (Gibco).

**Table 1:** Cell lines and culture conditions used during the study.

Cell line	Cell type	Origin	Growth conditions
Madin-Darby bovine kidney-MDBK*	Epithelial-like	Kidney of adult *Bos taurus* (Madin, 1958)	DMEM +10% FBS at 41°C—5% CO2
Cellosaurus LSCC-HD11*	Macrophage-like	Chicken, transformed with avian myelocytomatosis type MC29 virus (Beug et al., 1979)	RPMI +10% FBS at 41°C—5% CO2
Human squamous cell carcinoma cell line-COLO-680N **	Epithelial-like	Human squamous carcinoma cell line (Raida,1999)	DMEM +10% FBS at 37°C—5% CO2
Hamon Cancer Center 4006-HCC4006**	Epithelial-like	Lung adenocarcinoma (Gazdar, 2010)	DMEM +10% FBS at 37°C—5% CO2

* Previously tested with *E*. *tenella*; ** Not previously tested with *E*. *tenella*.

Twice weekly, cells were washed with phosphate-buffered saline (PBS), dissociated using trypsin-EDTA 0.25% (Gibco), washed with medium, centrifuged at 1200g/10min, suspended gently in medium and seeded at the optimal numbers for a confluence of 80% during regular passages. To keep their natural characteristics, cells were passed no more than 5 generations before reverting to a seed stock. The viability of cells was tested using the trypan-blue method before placing cells either in Chambered Coverslips μ-Slide 8 Wells (Ibidi) or Fisherbrand™ 96-Well Plates [26].

### 2.3. 2D culture

Monolayers of cells were seeded into each chamber well at 0.2×10^6^ cells/square two hours before infection, aiming for full confluence at the time sporozoites were added. Around 100μl of medium containing freshly purified sporozoites (0.6×10^6^; multiplicity of infection (MOI) of 1:3) were added to each well and cells incubated at 41°C—5% CO_2_. Two hours after infection, cells were gently washed twice with warmed PBS to remove extracellular sporozoites. The media was replaced every two days, and at least two independent experimental repetitions were performed in each system. Photographs were taken using a Leica DMI300 microscope equipped with a high-speed DCF365FX camera (Leica Microsystems, UK). Infected cells were identified by looking at intracellular sporozoites in evenly spaced fields using 20x or 40x objectives.

### 2.4. Collagen matrix-based cultures

Extracellular matrices were produced using two different protocols. The first used hydrogel ECM Gel from Engelbreth-Holm-Swarm murine sarcoma (Sigma) mixed at different proportions (30%, 50% or 70%) with culture media, following the manufacturer’s instructions. The second used bovine collagen I (Gibco) as extracellular matrix mixed 1:1 with media at concentrations of 3mg/ml and 5mg/ml according to manufacturer’s recommendations. ECM Gel and bovine collagen I were maintained at 15°C before experiments and quickly manipulated to avoid precocious polymerization.

#### 2.4.1. 2.5D

A thin layer of collagen matrix (100μl) prepared using each of the protocols described above was coated onto the bottom of each chamber square and left to polymerise at 37˚C for 30min. Each cell line (0.2×10^6^ cells/square) was resuspended in media and seeded on top of the collagen matrix and left to incubate at 41˚C with 5% CO_2_ for 1 hour. After this period, non-attached cells were washed and freshly-purified sporozoites were added/washed, as described in section 2.3.

#### 2.4.2. Collagen (3D)

A thin layer of collagen matrix (100μl) prepared using either protocol described above was coated onto the bottom of the chamber slide and left to polymerise at 37˚C for 30min. Cells were prepared at a concentration of 0.2×10^6^ in 150μl and mixed with 150μl of the matrix (ECM Gel or Bovine Collagen I). The mix was left at 37˚C for 30 minutes to polymerise. Cell viability was checked again, considering the need for manipulation of either the ECM Gel or Bovine Collagen I at low temperatures(<20˚C). Chambers were incubated at 41˚C—5% CO_2_ for 2 hours before infection. Infections were performed using the same sporozoite doses as in the 2D and 2.5D systems, except that sporozoites here were washed at either 2 or 14 hours post infection (hpi).

### 2.5. Spheroids (3D)

Scaffold-free 3D cell spheroids were generated using the liquid overlay technique (LOT) as described by Metzger (2011). Every cell line included in the study was tested using different numbers of cells (50, 100, 1000 or 5000) in 200μl DMEM per well to optimise the number of cells/well to generate stable spheroids for the study period. Cells were placed in Fisherbrand™ 96-Well Plates coated with 50μL 1% sterile agarose gel. Plates were incubated at 41˚C—5% CO_2_ and checked for five days for the spheroid’s formation and stability. Infection of spheroids with *E*. *tenella* was performed as described in section 2.3. A MOI of 1:3 was tested following a period of at least 10 days using 100 cells/well.

### 2.6. Analysis

Reported results were based on observations of at least two repeated infections per system for each cell line. The diameter of cells was measured by ImageJ distribution Fiji, using photographs of non-infected cells in suspension. Sporozoites were counted as being intracellular when the post-invasion merger of the anterior and posterior refractile bodies (RB) could be clearly seen [27]. After normality tests, the comparison of groups was analysed using the Kruskal-Wallis test, followed by Dunn’s multiple comparison test. Correlation was performed using the Spearman test. All statistical analysis was done using the software GraphPad Prism 9.

## 3. Results

### 3.1. Schizont development was supported by all cell lines tested in 2D culture

**Fig 1:**
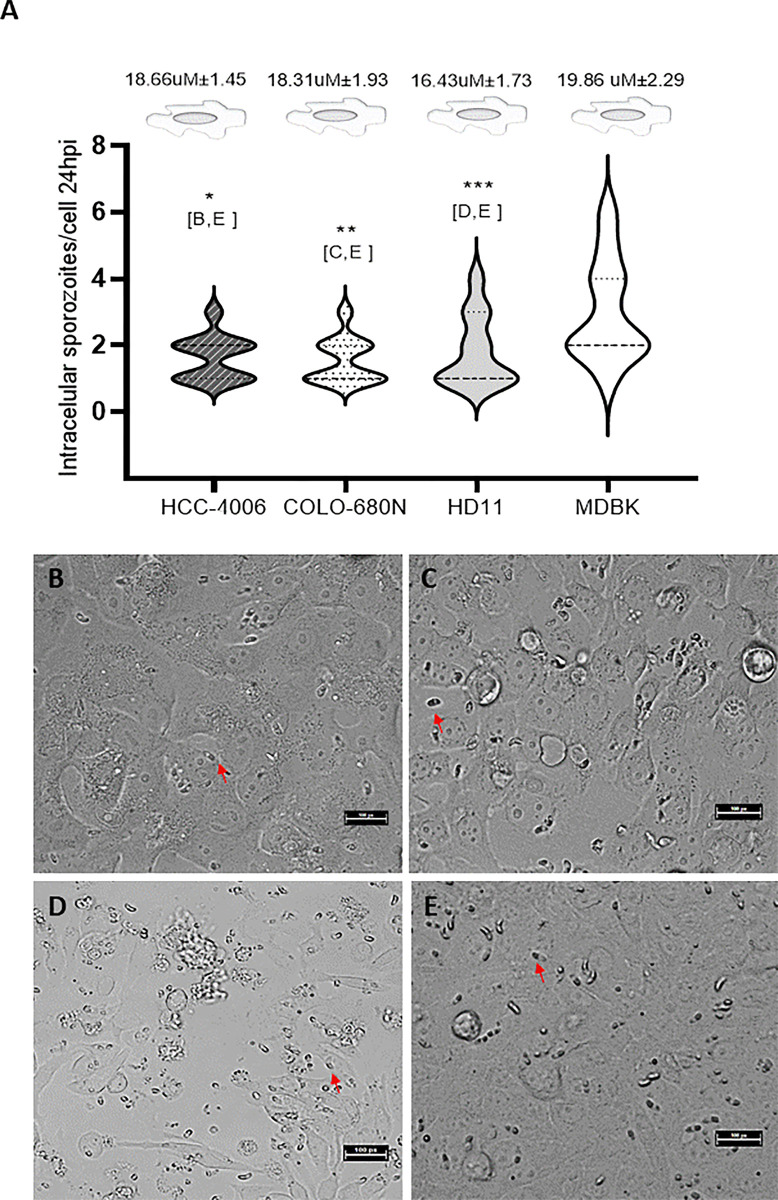
Development of *E*. *tenella* at 24 hours post infection (hpi) in 2D systems. A. The violin plot represents the number of sporozoites showing RB merger per cell. Lines in each plot represent the median and quartiles. The average diameter ± standard deviation for each uninfected cell line is shown at the top. B-E. Intracellular sporozoites in HCC-4406 (B), COLO-680N (C), HD11 (D) and MDBK (E) cells. Red arrows indicate sporozoites with RB merger supporting the intracellular location. Scale-bars: 25 μm. Letters in brackets indicate pairs with significant difference (Dunn’s multiple comparisons test), *p < 0.05; **p < 0.01.

*Eimeria tenella* intracellular sporozoites were seen from 2hpi in all cell lines, including first reports for *Eimeria* invasion of the human cell lines COLO-680N and HCC4006. The level of cell invasion measured as the number of sporozoites containing a single (merged) RB 24hpi/cell varied significantly among the cell lines tested (Kruskal-Wallis test, p<0.05) (Fig 1). MDBK cells showed a significantly higher number of intracellular sporozoites compared to the other cell lines (Dunn’s multiple comparisons test, p<0.05 MDBK vs HCC-4406, COLO-680N and HD11). Significant cell monolayer destruction was observed for HD11 monolayers despite using the same MOI in all cell lines (Fig 1D). Cells of the HD11 line were smaller than those from the other cell lines, so we investigated if the diameter of cells could be a factor related to monolayer destruction; however, the correlation between the number of intracellular sporozoites and cell diameter did not support this hypothesis (Spearman test, p<0.05, r = 0.2866).

**Fig 2:**
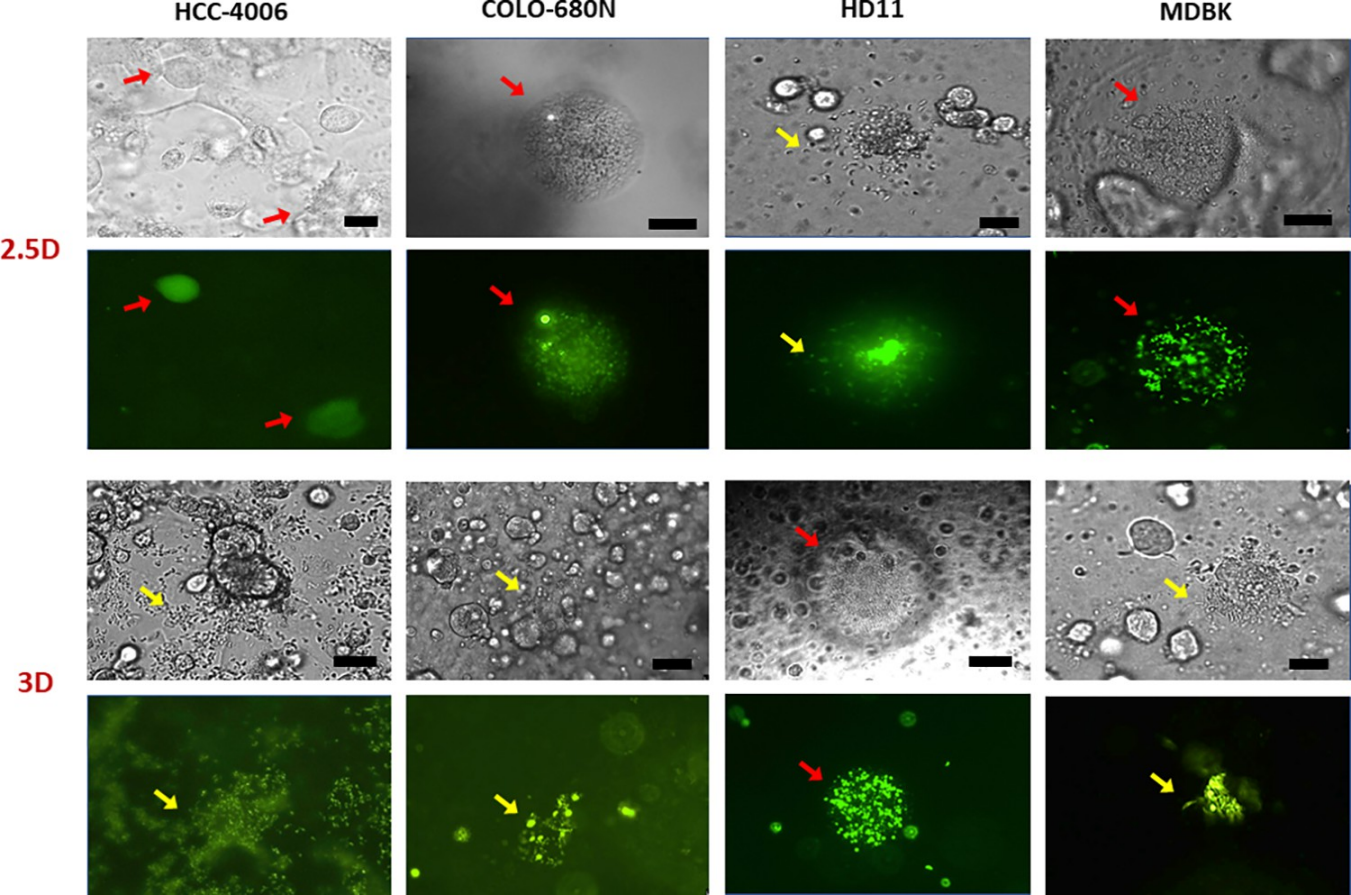
Cell lines infected with *E*. *tenella* using 2.5D and 3D cell systems on ECM Gel from Engelbreth-Holm-Swarm murine sarcoma. Cell line identity is indicated at the top of the panels; cell system is indicated on the left. The top panels (per system) show a bright field with the corresponding fluorescent fields below. Red arrows point to developed schizonts, and yellow arrows indicate hatched merozoites. Scale-bars: 25 μm.

Use of the Et-dYFP population allowed direct observation of sporozoites and their development by fluorescent microscopy. Comparable profiles of development were seen among all cell lines tested (Fig 2). At 48hpi, schizonts were observed in all cell lines at similar levels of development. The presence of schizonts and the release of merozoites was seen intensively until 72hpi; after this period, smaller schizonts were observed and no clear morphological forms indicated the presence of sexual stages. After ten days, some sporozoites were still observed undeveloped inside cells (in all cell lines) with the same initial level of fluorescence.

### 3.2. Collagen matrix-based cultures (2.5D and 3D)

Using the first protocol (ECM gel) the initial test for stability indicated that cells were maintained on the matrix for 8 days, after which all cells had migrated to the bottom of the chamber completely. MDBK cells in particular showed migration from 12 hours after seeding in the 2.5D and 3D systems, but with greater intensity after 8 days. A monolayer was formed on top of the matrix in the 2.5D system with cells that remained on top, sometimes forming cell clumps. Due to the migration of cells to the bottom of the chamber, a second monolayer was very often seen on the bottom of the chamber 24h post seeding. The migration of HD11 was slowest, but was complete in all matrix: media ratio mixes by 24 hours after seeding in the 2.5D system.The thicker layer of ECM in the 3D system appeared to reduce cell migration and this was independent of the ECM concentration used. Cell viability was lower in the 3D system with ~30–50% of cell loss, in contrast to 2D or 2.5D systems with <5% of cell loss; this may be due to cells being manipulated at lower temperatures when mixing with the matrix

Invasion of sporozoites in the 2.5D system was observed for all cell lines from 2hpi. No sporozoite invasion was observed in the 3D system when parasites were washed at 2hpi, but infection was observed throughout the ECM if sporozoite washes were delayed until 14hpi. At 24hpi it was possible to observe different levels of intracellular invasion depending on the depth of the matrix (in all cell lines). Cells seeded on the top (2.5D) or inside the matrix (3D) showed lower infection in comparison to those that migrated to the bottom of the chamber. Cells located at the top (2.5D) or inside (3D) the matrix were seen carrying a maximum of one or two sporozoites per cell. High concentrations of ECM (>70%) did not impair or reduce the penetration of sporozoites into the matrix and cells since similar sporozoite numbers were seen invading all cell lines and concentrations at 24hpi. After 48hpi the presence of round fluorescent schizonts were seen in all cell lines together with released merozoite (Fig 2). During the following days, merozoites and smaller schizonts were observed in all cell lines. After 8 days, there was an intense migration of cells to the bottom of chamber, but still some schizonts/merozoites were seen. Some intracellular sporozoites did not develop to further stages and some we found suspended in extracellular matrix still showing fluorescence (potentially alive).

Bovine Collagen I was tested as a possible alternative to ECM gel to produce improved matrix quality. Using this protocol there was a slight reduction in cell migration to the bottom observed on day 8 and an overall improvement in cell viability in the 3D system (>70%). However, a similar level of *E*. *tenella* sporozoite infections and development to that achieved in ECM was observed in all tested cell lines.

### 3.3. Spheroids

**Fig 3:**
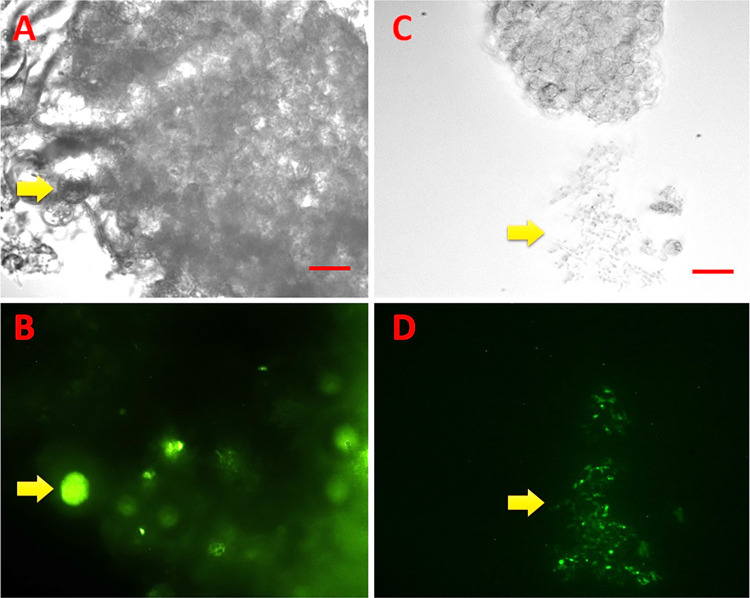
HD11 spheroids three days after infection with Et-dYFP sporozoites. A-B. Infected spheroid showing a mature schizont (yellow arrow) C-D. Infected spheroid showing released merozoites (yellow arrow). A, C. bright field; B, D. fluorescent filter. Scale-bars: 25 μm.

Only HD11 cells successfully produced spheroid structures, growing in numbers and remaining detached from the bottom for five days. Spheroid formation was observed 24 hours after induction at which point these 24-hour spheroids (100 cells/well) were used for sporozoite infections. Parasite invasion and development inside the spheroids was confirmed by the release of merozoites two days after infection of the HD11 spheroids (Fig 3A) and the presence of schizonts was clearly observed in spheroid borders. In line with the 2D, 2.5D and 3D culture systems described above, no further developmental stages were observed beyond first generation merozoites in this model. However, observation of intracellular parasites was difficult due to high host cell concentration at the central area of the spheroid. *Eimeria tenella* merozoites recovered from the spheroid media three days after initial infection were placed into fresh 2D monolayers. We found these merozoites invaded new HD11 cells (fluorescence was present inside the cell) but there was no visualization of further later stages over the following six days.

## 4. Discussion

Challenges posed by the inability to efficiently culture *E*. *tenella* through its complete lifecycle *in vitro* have long been recognised, hindering understanding of biological function and development of novel controls measures. To date, only primary chick kidney cells and the immortalised cell line CLEC-213 –derived from chicken lung epithelium–has supported completion of the entire *E*. *tenella* life cycle, but at very low efficiencies and for CLEC-213 requiring use of a precocious parasite line that undergoes a single round of schizogony (11). Low efficiency and continued need to sacrifice animals to source oocysts/sporozoites have restricted use of these approaches, with studies of parasite growth *in vitro* limited to ‘semi-cultivation’ in 2D systems using immortalised cell lines [28]. This contrasts with significant advances in the cultivation of apicomplexan parasites that can cause human diseases such as species of *Plasmodium* and *Cryptosporidium*, where novel techniques and resources have been applied resulting in successful establishment of *in vitro* life cycles [21, 29].

In 2D cultures, *E*. *tenella* invaded and developed through the first round of schizogony in all four cell lines tested in this study, including two human-derived lines not previously tested. These two were selected because they support the further development of *Cryptosporidium parvum* [30]. Comparing invasion rates for the four tested cell lines indicated that MDBK was most efficient, confirming that this host cell remains a good option to study early parasite development when compared to other immortalized cell lines [29].Visualization of sporozoites showing RB merger is a signature of intracellular location [27], so we used this simple marker as a method to assess invasion as it is reliable and simple compared to other methods that require sample fixation and staining. The HD11 cell line was most disrupted by *E*. *tenella* infection; at the same MOI as the other cell lines, monolayer damage was seen from 24hpi although this did not limit the capacity of sporozoites to develop further within those cells that remained. HD11 is derived from the chicken and has been reported to sustain *E*. *tenella* infections *in vitro* [31]. Recently, [32] suggested that HD11 sustained *E*. *tenella* replication to the same degree or better than MDBK cells. The macrophage-like rather than epithelial nature of this cell line could be related to the weaker monolayer resistance observed here after the infection. In addition, different responses of cell lines for wounding repair after being traversed by sporozoites should be considered. This mechanism is well described in apicomplexan models, but little explored in *Eimeria* infections using different cell lines such as HD11 [33].

We used a transgenic *E*. *tenella* population that has been modified to express a double copy of the YFP fluorescent reporter, permitting rapid visualisation of sporozoites and subsequent stages of parasite development. The *in vitro* development of this transgenic population in MDBK cells has been shown to be equivalent to the parental *E*. *tenella* Wisconsin strain [34]. Here, similar parasite development was observed among all studied cell lines. However, these results are qualitative findings intended to evaluate development beyond first-generation schizonts; occurrence of schizonts and merozoites was not assessed by quantitative methods. Our results in the 2D system are in accordance with previous studies where first-generation merozoites were observed but failed to progress further.

Host cellular morphology significantly differed when grown in 3D and 2.5D. In the former system, cells from all cell lines acquired a spherical shape. An intermediate shape was observed in the 2.5D system, with cell clusters and monolayers formed on the top of matrix. In the 3D system, sporozoites were able to penetrate even the high concentrations (up to 70%) of collagen matrix-based cultures and infect cells, an interesting observation considering that *T*. *gondii* tachyzoites did not penetrate the matrix at higher concentrations [22]. However, sporozoites needed extra time to penetrate the matrix and invade cells in the 3D system, as evidenced by the lack of invasion if sporozoites were washed away at 2hpi. This is reasonable considering the high viscosity of the physical barrier created that sporozoites needed to traverse [35]. Stable cell suspensions in the collagen matrix were not achieved for any cell lines tested and most sporozoites, schizonts and merozoites were observed in cells at the bottom, although it was not determined whether infection occurred before or after migration. When cells were tested in suspension using very high concentrations of collagen matrix (80% and 90%) in an attempt to prevent cell migration, sporozoites did not infect at all.

We were able to generate spheroids only for HD11 cells; a non-epithelial cell line (macrophage derived) with weaker adherence properties that favours building a scaffold-free cell culture model [36]. Sporozoites infected HD11 spheroids and inside the non-adhered cells schizonts adopted a spherical shape similar to those seen in the collagen matrix systems or the recently created scaffold-free 3D model of enteroids [24].This system also showed some limitations as after five days cells started to multiply and adhere to the bottom, restricting model functionality and visualisation of parasites.

An evident variation between the 3D (ECM or bovine collagen matrix/spheroid) and 2D systems tested here was the influence on the morphology of intracellular forms of *E*. *tenella*. In 2D monolayers, schizonts encompassed the shape of cells, which were polarized at the bottom of chambers; whereas schizonts in non-attached cells showed a more spherical shape (S1 Fig). Similar findings were described by [22] when attempting to grow *T*. *gondii* in a collagen matrix system. Advances in 3D culture systems offer conditions that are more similar to natural hosts [37] but are compromised in 2D systems. This study has explored for the first time the use of collagen-based matrix and spheroid systems using new and established cell lines with different characteristics to study *E*. *tenella* infection. The parasite has successfully infected cells and developed to first-generation merozoites, however, further development has not been observed.

## Supporting information

S1 Fig Madin-Darby bovine kidney cells cultured using different systems infected with *E*. *tenella*. Left-side pictures show bright field and the corresponding fluorescent field of a schizont in the 2D system. Right-side pictures show bright field and the corresponding fluorescent field of a schizont popping out from an infected cell in the 3D system. Scale-bars: 25 μm.
(TIF)

S2 Fig Number of intracellular sporozoites per cell lines observed 24 hours post invasion. (TIF)
