# Supplementary figures and images for: Comparative study of Eimeria tenella development in different cell culture systems
Source: PLoS One. 2024 Jul 18;19(7):e0307291. doi: 10.1371/journal.pone.0307291 (PMC11257319; doi:10.1371/journal.pone.0307291)

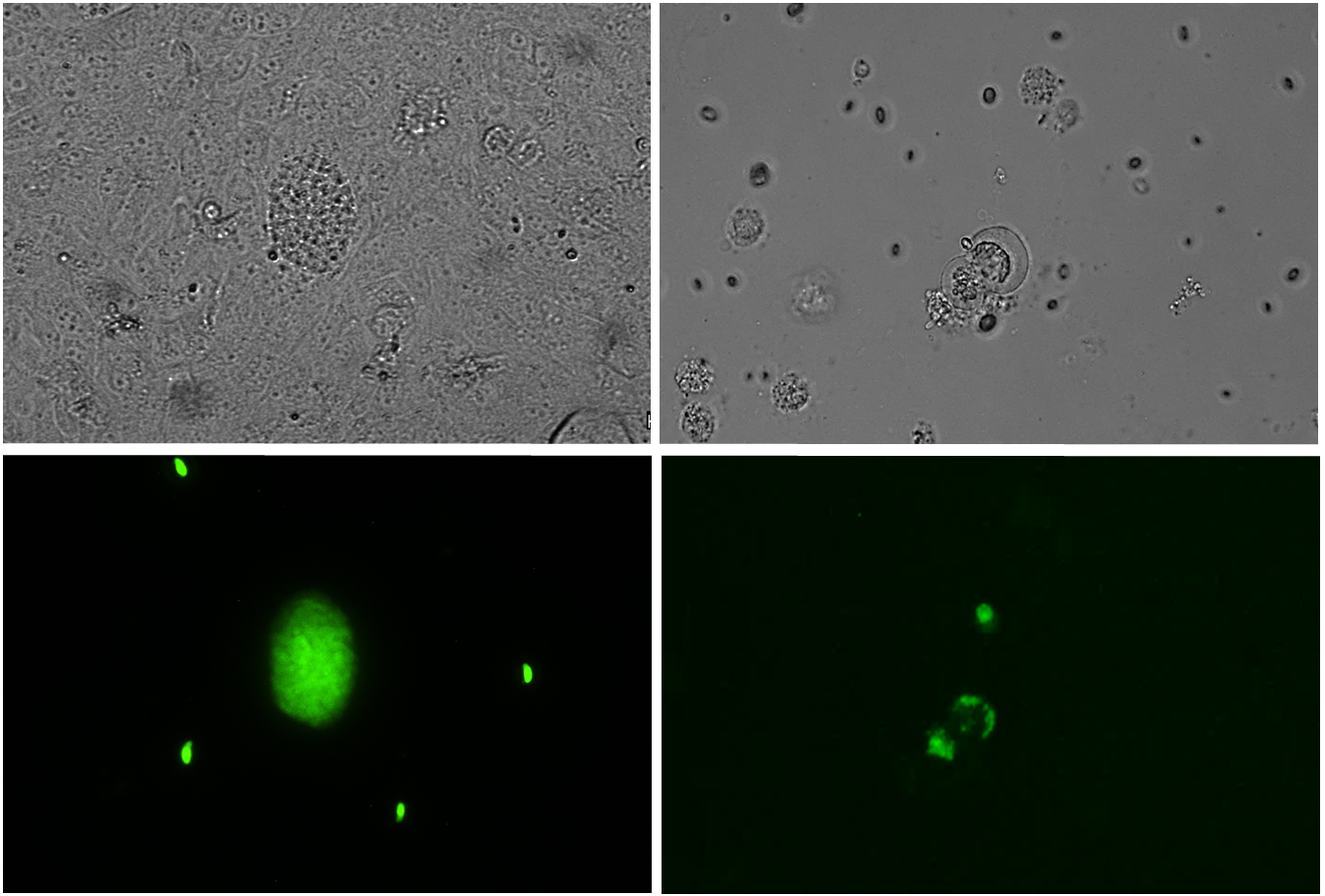

Supplement: S1 Fig — Left-side pictures show bright field and the corresponding fluorescent field of a schizont in the 2D system. Right-side pictures show bright field and the corresponding fluorescent field of a schizont popping out from an infected cell in the 3D system. Scale-bars: 25 μm. (TIF) [file pone.0307291.s001.tif]

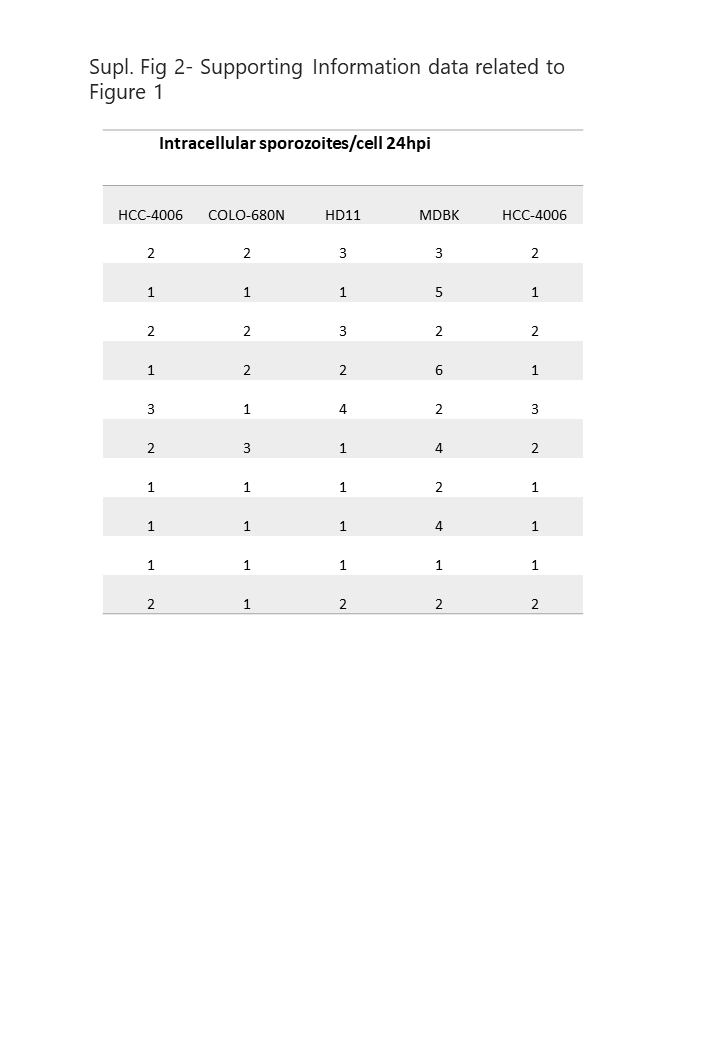

Supplement: S2 Fig — (TIF) [file pone.0307291.s002.tif]
